# De novo thymic carcinoma or malignant transformation: a myasthenic patient presented with multiple mediastinal tumours

**DOI:** 10.1002/rcr2.629

**Published:** 2020-07-23

**Authors:** Chu‐Pin Pai, Chih‐Ming Lin, Yi‐Chen Yeh, Chien‐Sheng Huang, Biing‐Shiun Huang

**Affiliations:** ^1^ Division of Thoracic Surgery, Department of Surgery Taipei Veterans General Hospital Taipei Taiwan; ^2^ Department of Pathology Taipei Veterans General Hospital Taipei Taiwan; ^3^ Institute of Clinical Medicine, School of Medicine National Yang‐Ming University Taipei Taiwan

**Keywords:** Extended thymectomy, myasthenia gravis, thymoma, thymic carcinoma

## Abstract

A 63‐year‐old man presented with bilateral ptosis, and detailed evaluation confirmed ocular myasthenia gravis with three anterior mediastinal masses on computed tomography (CT) of the chest. Extended thymectomy was performed, and pathology revealed two thymic carcinoma and one thymoma. After surgery, the patient is free from recurrence. Synchronous triple thymic carcinomas and thymoma have not been reported. The finding of this case report supports the hypothesis of malignant transformation of thymoma to thymic carcinoma. Thymic carcinoma should be considered in the differential diagnosis of multiple thymic tumours, and extended thymectomy should be the treatment of choice.

## Graphical abstract

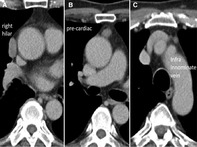

## Introduction

Thymic carcinoma may arise de novo, as well as develop from malignant transformation from thymoma. Given the potential of malignant change, it is unclear whether patients with multiple thymoma would have a higher chance of synchronous thymic carcinoma. There have been previous reports of mixed thymoma and thymic carcinoma within the same lesion. However, no isolated thymoma and thymic carcinoma have been reported. We discuss the development of thymic carcinoma, and its association with synchronous thymoma.

## Case Report

**Figure 1:**
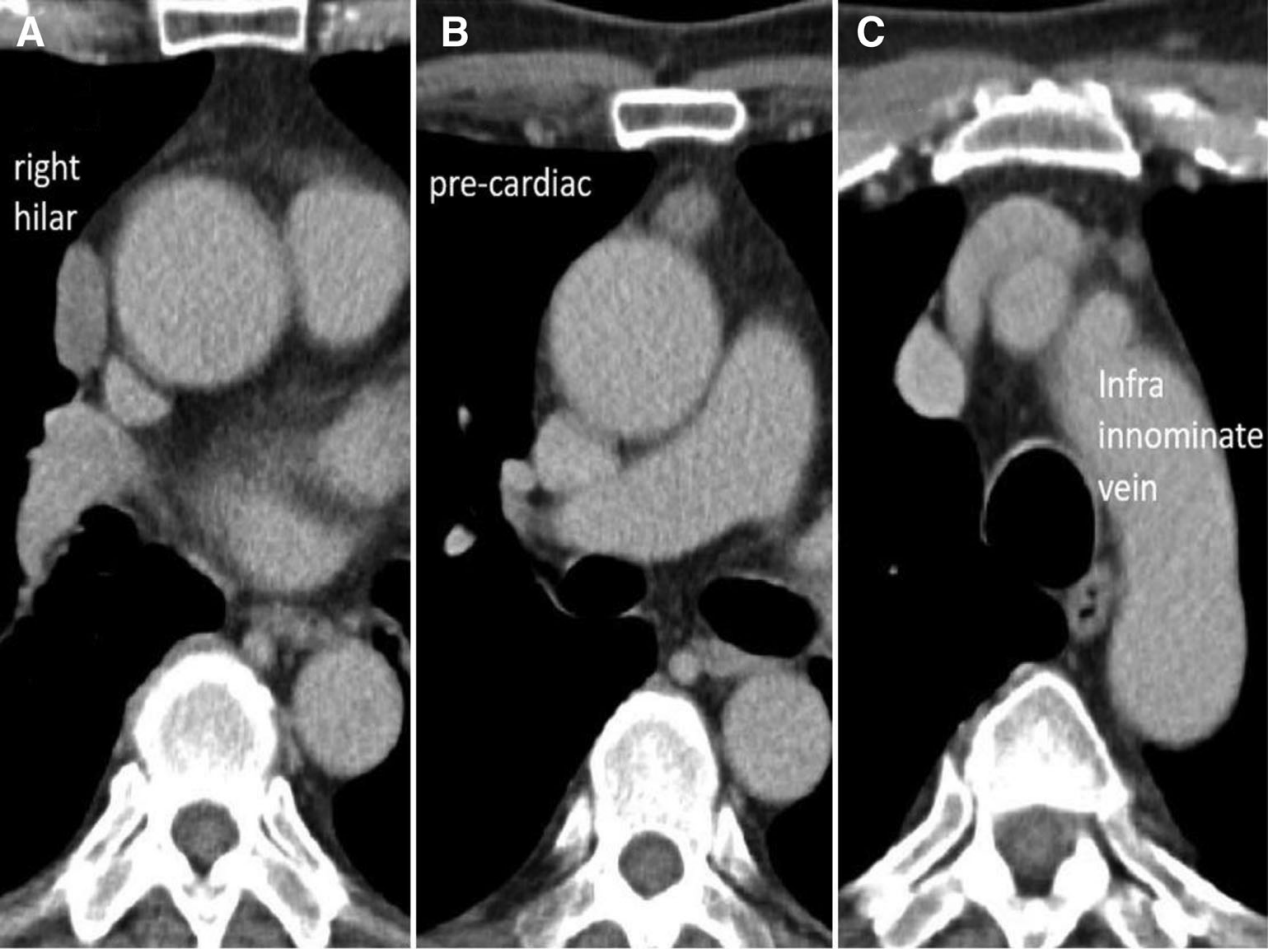
Three separate anterior mediastinal nodules located at the right hilar (A, 2.3 × 1.2 cm), pre‐cardiac (B, 1.4 × 0.9 cm), and infra‐innominate vein space (C, 0.6 × 0.5 cm), respectively. The three anterior mediastinal nodules were all round or ovoid in shape and were smooth in contours and there were no enlarged lymph nodes nearby identified. Two thymic tumours (A and B) with a non‐specific small lymph node (C) was impressed preoperatively in the present case.

**Figure 2:**
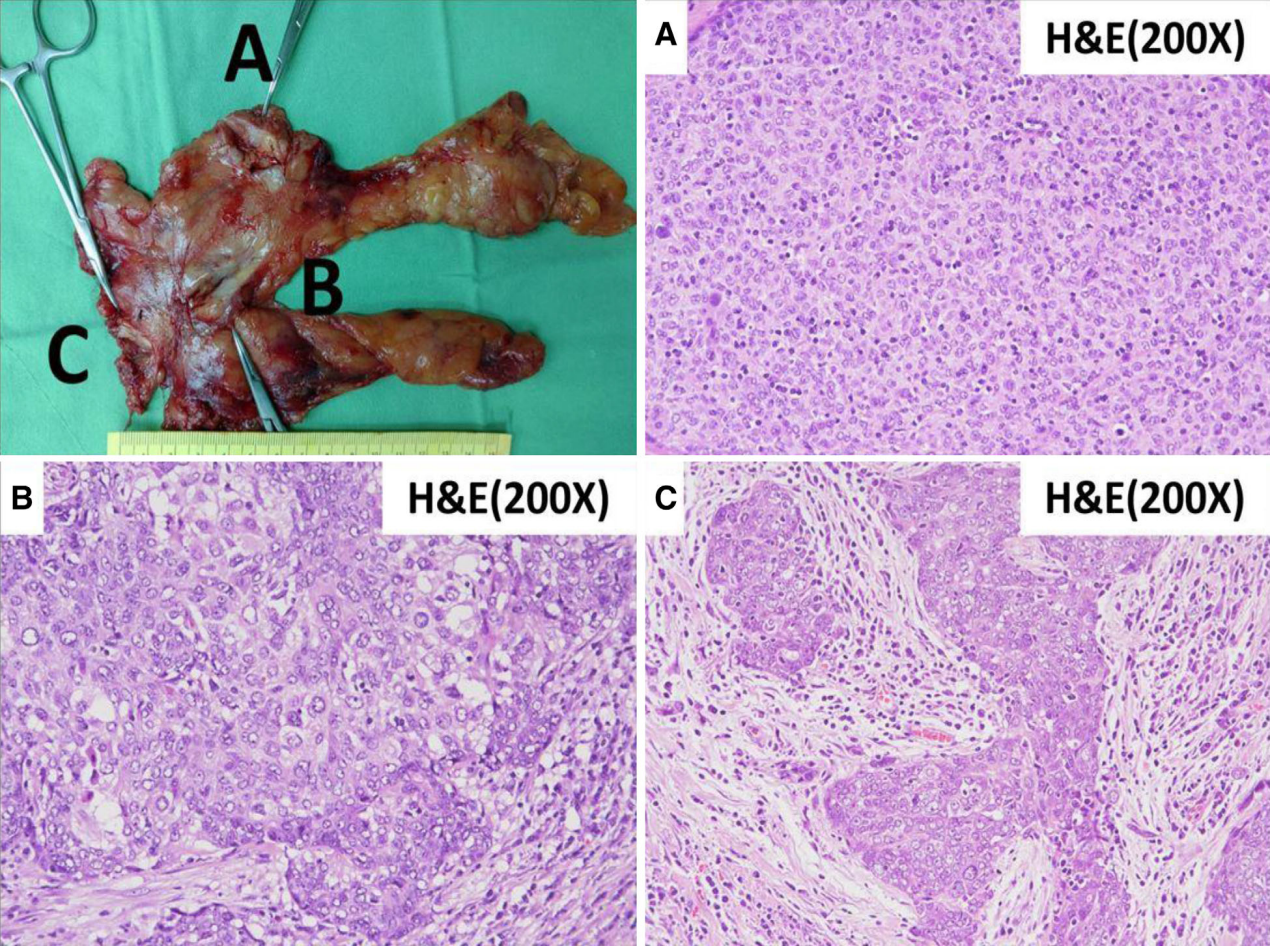
Grossly, three separate nodules were found in the extended thymectomy specimen. (A) Microscopically, sections of tumour A show picture of thymoma, which are immunoreactive for p40, and non‐reactive for CD5 and CD117 with scattered terminal deoxynucleotidyl transferase (TdT)‐positive lymphocyte inside the tumour. (B, C) Sections of both tumour B and tumour C show squamous cell carcinoma, non‐keratinizing, which are immunoreactive for p40, focally immunoreactive for CD5 and CD117, with negative immunostaining for terminal deoxynucleotidyl transferase (TdT) in the tumour.

We present a 63‐year‐old male diagnosed as ocular type myasthenia gravis (MG) and transferred for surgical intervention of suspicious mediastinal tumours (Fig. 1; three separate anterior mediastinal nodules located at the right hilar (2.3 × 1.2 cm), pre‐cardiac (1.4 × 0.9 cm), and infra‐innominate vein space (0.6 × 0.5 cm), respectively). Preoperative serum anti‐acetylcholine receptor antibody level was elevated (1.1; normal level < 0.5 nmol/L). After discussion, bilateral video‐assisted thoracoscopic extended thymectomy was performed and grossly three separate soft nodules were found in the resected thymic specimen (Fig. 2). Pathology revealed one thymoma, World Health Organization (WHO) type B3, modified Masaoka stage IIA with microscopic transcapsular invasion located at the right hilar region (tumour A), and two isolated squamous cell carcinoma, non‐keratinizing, modified Masaoka stage IIA with microscopic transcapsular invasion located at the pre‐cardiac (tumour B) and the infra‐innominate vein space (tumour C). The resection margin was 0.1 cm for tumour A, and free for both tumours B and C. Post‐operative adjuvant radiation therapy with 6000 cGy/30 fractions was prescribed. The patient is still taking pyridostigmine 60 mg three times a day and no image evidence of recurrence noted for 20 months after the operation.

## Discussion

Multiple thymoma is rare, with incidence ranging from 1.1% to 3.1% in previous literature [1]. To our knowledge, synchronous triple separated thymic carcinomas and thymoma have not been reported. It is controversial whether cases of multiple thymomas represent multicentric origin or intra‐thymic metastasis. The characteristics which are in favour of multicentric origin rather than intra‐thymic metastasis included limited number of tumours (less than three), similar sizes, and early in stage [1, 2]. In contrast, similar histological findings in pathology would support intra‐thymic metastasis. In our case, three variable sized tumours were all not in early stage and thus had the potential to metastasize.

On the basis of previous observation of thymic epithelial tumours that harbour both squamous cell carcinoma and conventional (usually B3) thymoma, Suster and Moran suggested that thymic squamous cell carcinoma can arise from pre‐existing thymoma after malignant transformation or progressive loss of differentiation [3]. This hypothesis is further confirmed by the reported evidence of histological progression when recurrence occurs within the cortical histological differentiation [4]. Notably, the present case also supports this hypothesis through the presentation of synchronous isolated multiple thymic tumours with the existence of a continuum in the spectrum of differentiation between thymoma and thymic carcinoma. Moreover, although the two thymic carcinomas were smaller than the B3 thymoma, they harbour higher malignant potential. Therefore, intra‐thymic metastases of thymoma followed by malignant transformation into the thymic carcinoma should be considered.

In the majority of cases, preoperative radiological features and clinical presentation may suggest the clinical diagnosis of thymoma from that of thymic carcinoma, with approximately 45% of the former have MG, and the latter often have irregular margins and enlarged lymph nodes, and are rarely diagnosed in patients with MG [5]. The preoperative clinical impression was the presence of two thymomas (A and B) and a non‐specific small lymph node (C); however, the final histology proved this to be not the case, and therefore leading to the debatable question of the extent of excision for thymoma. Based on the experience of our case and to minimize the possibility of tumour recurrence, extended thymectomy should be the treatment of choice for patients with mediastinal tumour even without the expectation of ectopic thymic carcinoma.

In conclusion, despite preoperative image favouring multiple thymoma, thymic carcinoma should be considered in the differential diagnosis due to possible malignant transformation, and extended thymectomy should be the treatment of choice for the same reason to minimize the chance of recurrence.

### Disclosure Statement

Appropriate written informed consent was obtained for publication of this case report and accompanying images.
